# Association between stopping universal SARS-CoV-2 admission testing and hospital-onset SARS-CoV-2 in England and Scotland

**DOI:** 10.1017/ash.2023.391

**Published:** 2023-09-29

**Authors:** Theodore Pak, Chanu Rhee, Michael Klompasl

## Abstract

**Background:** Many hospitals test all patients for SARS-CoV-2 upon admission to prevent silent transmission to other patients and healthcare workers. The utility of universal admission testing has been questioned, however, due to resource constraints, care delays, and sparse data on its impact on nosocomial infections. England and Scotland stopped requiring universal admission testing on August 31, 2022, and September 28, 2022, respectively. We assessed associations between these changes and hospital-onset SARS-CoV-2 infection rates. **Methods:** We used public data from National Health Service England and Public Health Scotland on hospital-onset SARS-CoV-2 infections, defined as cases diagnosed >7 days after admission, between July 1, 2021, and December 16, 2022. Because hospital-onset infections are driven by SARS-CoV-2 community incidence rates, we calculated the weekly ratio between hospital-onset versus community-onset SARS-CoV-2 admissions (diagnosed ≤7 days from admission) and assessed for temporal changes associated with stopping universal admission testing using interrupted time-series analysis. The study was divided into 3 periods: sARS-CoV-2 delta-variant dominance with admission testing, SARS-CoV-2 omicron-variant dominance with admission testing (starting December 14, 2021), and SARS-CoV-2 omicron-variant dominance without admission testing. **Results:** During the study period, there were 518,379 COVID-19 admissions in England, including 398,264 community-onset and 120,115 hospital-onset cases, and 46,517 COVID-19 admissions in Scotland, including 34,183 community-onset and 12,334 hospital-onset cases. The mean weekly ratio of new hospital-onset SARS-CoV-2 infections versus community-onset admissions in England rose from 0.12 during the SARS-CoV-2 delta-variant surge to 0.33 during the SARS-CoV-2 omicron-variant surge to 0.48 after universal admission testing ended (Fig.). There was a significant immediate level change both after the SARS-CoV-2 delta-to-omicron variant transition (92% relative increase; 95% CI, 58%–127%) and after admission testing ended (32% relative increase; 95% CI, 14%–50%). Likewise, the mean weekly ratios rose from 0.11 to 0.43 to 0.89 during their analogous periods in Scotland, with significant level changes both after SARS-CoV-2 delta-to-omicron variant transition (113% relative increase; 95% CI, 54%–172%) and after admission testing ended (72% relative increase; 95% CI, 43%–100%). No significant trend changes were observed. **Conclusions:** Stopping asymptomatic screening of hospitalized patients in 2 national health systems was associated with significant increases in hospital-onset SARS-CoV-2 infections. Nosocomial SARS-CoV-2 remains a common and potentially morbid complication, with reported mortality rates for nosocomial infections by SARS-CoV-2 omicron variant ranging from 5% to 13%. Preventing infections in vulnerable populations remains an important safety goal. Hospitals should exercise caution when considering reductions in SARS-CoV-2 admission screening.

**Figure:**
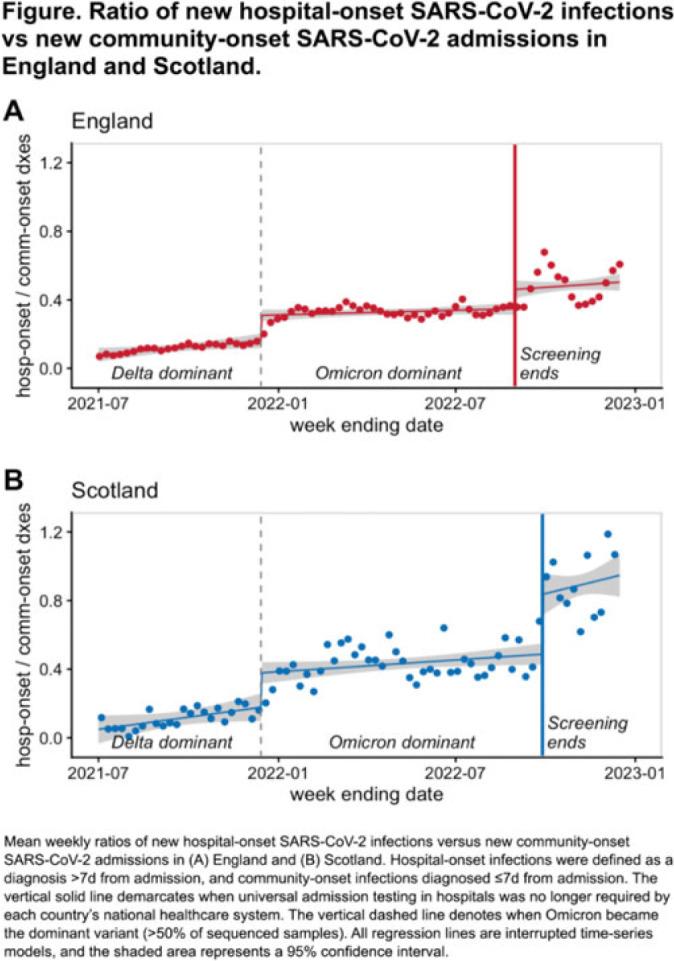

**Disclosures:** None

